# Simvastatin Impairs Glucose Homeostasis in Mice Depending on PGC-1α Skeletal Muscle Expression

**DOI:** 10.3390/biomedicines8090351

**Published:** 2020-09-15

**Authors:** Miljenko Valentin Panajatovic, François Singh, Stephan Krähenbühl, Jamal Bouitbir

**Affiliations:** 1 Division of Clinical Pharmacology & Toxicology, University Hospital, 4031 Basel, Switzerland; m.panajatovic@unibas.ch (M.V.P.); fzsingh@dundee.ac.uk (F.S.); stephan.kraehenbuehl@usb.ch (S.K.); 2 Department of Biomedicine, University of Basel, 4031 Basel, Switzerland; 3 Swiss Center for Applied Human Toxicology, 4031 Basel, Switzerland

**Keywords:** simvastatin, PGC-1α, iGTT, insulin, glucose uptake, Glut4

## Abstract

Several studies showed an increased risk for diabetes with statin treatment. PGC-1α is an important regulator of muscle energy metabolism and mitochondrial biogenesis. Since statins impair skeletal muscle PGC-1α expression and reduced PGC-1α expression has been observed in diabetic patients, we investigated the possibility that skeletal muscle PGC1α expression influences the effect of simvastatin on muscle glucose metabolism. Mice with muscle PGC-1α knockout (KO) or PGC-1α overexpression (OE), and wild-type (WT) mice were investigated. Mice were treated orally for 3 weeks with simvastatin (5 mg/kg/day) and investigated by intraperitoneal glucose tolerance (iGTT), in vivo skeletal muscle glucose uptake, muscle glycogen content, and Glut4 and hexokinase mRNA and protein expression. Simvastatin impaired glucose metabolism in WT mice, as manifested by increased glucose blood concentrations during the iGTT, decreased skeletal muscle glucose uptake and glycogen stores. KO mice showed impaired glucose homeostasis with increased blood glucose concentrations during the iGTT already without simvastatin treatment and simvastatin induced a decrease in skeletal muscle glucose uptake. In OE mice, simvastatin treatment increased blood glucose and insulin concentrations during the iGTT, and increased skeletal muscle glucose uptake, glycogen stores, and Glut4 and hexokinase protein expression. In conclusion, simvastatin impaired skeletal muscle insulin sensitivity in WT mice, while KO mice exhibited impaired skeletal muscle insulin sensitivity already in the absence of simvastatin. In OE mice, simvastatin augmented muscular glucose uptake but impaired whole-body insulin sensitivity. Thus, simvastatin affected glucose homeostasis depending on PGC-1α expression.

## 1. Introduction

Statins, the most often prescribed lipid-lowering drugs, are highly effective for prevention and treatment of cardiovascular diseases associated with dyslipidemia [1,2]. Statins impair cholesterol synthesis by inhibiting 3-hydroxy-3-methylglutaryl coenzyme A reductase (HMG-CoA reductase), which stimulates LDL-C uptake by hepatocytes [3]. Although statins are generally well tolerated, they can lead to adverse effects mainly in skeletal muscle. The clinical manifestations range from myalgia and asymptomatic elevation of creatine kinase to potentially fatal rhabdomyolysis, leading to death in rare cases [4,5,6,7]. Myopathy affects up to 30% of the patients treated with statins, depending on the definition of myopathy and the patient population included [8,9,10]. The mechanisms by which statins induce myopathy are not fully understood. Several studies from our group have shown that mitochondrial dysfunction is an important component of statin-associated skeletal muscle damage [7,11,12,13]. These mitochondrial defects increase mitochondrial reactive oxygen species (ROS) production, which partly accounts for apoptosis of muscle fibers and promotes fiber degeneration and remodeling [11,13,14].

In addition to myopathy, long-term statin treatment is associated with insulin resistance, leading to an increased risk for diabetes. Large scale randomized controlled trials have demonstrated that statin treatment can induce hyperglycemia and augments the risk for diabetes [15,16]. For atorvastatin and simvastatin, it has been reported that they increase the risk for diabetes in a dose-dependent manner [17,18]. In populations with high risk for type 2 diabetes, approximately 30% of the patients treated with statins developed diabetes [19,20]. To date, the exact mechanism of the statin-induced insulin resistance is not entirely clear. We have demonstrated previously that simvastatin-induced insulin resistance and decreased glucose uptake into skeletal muscle of wild-type mice due to impaired translocation of Glut4 into the sarcolemma [21].

Peroxisome proliferator activated receptor gamma coactivator-1 alpha (PGC-1α) is a protein that can bind to certain transcription factors and thereby increases their capacity to induce target gene expression. An important characteristic of PGC-1α is its ability to stimulate mitochondrial biogenesis and to increase oxidative metabolism [22]. Impaired mRNA and protein expression of PGC-1α has been observed in many disease states. For instance, mRNA expression of PGC-1α, PGC-1β, and genes encoding for proteins involved in mitochondrial oxidative phosphorylation (OXPHOS) are reduced in skeletal muscle of patients with type 2 diabetes [23]. PGC-1α dysregulation has also been described in pre-diabetic individuals, suggesting that impaired PGC-1α expression could represent an early event in the development of type 2 diabetes [24]. In addition, we have shown previously that statins impair PGC-1α expression and mitochondrial biogenesis in skeletal muscle of patients, rats [11] and mice [13].

Based on these considerations, skeletal muscle PGC-1α expression could modify the effects of statins on skeletal muscle glucose metabolism. To study this question, we performed an intraperitoneal glucose tolerance test (iGTT) in wild-type mice (WT), PGC-1α muscle knock-out mice (KO mice), and in mice over-expressing PGC-1α (OE mice). Mice were treated with simvastatin for three weeks at 5 mg/kg by oral gavage. We also investigated the skeletal muscle glucose uptake in vivo and the skeletal muscle glycogen stores and Glut4 and hexokinase mRNA and protein expression. We hypothesized that simvastatin treatment affects skeletal muscle glucose homeostasis in WT and KO mice, while mice with PGC-1α over-expression are protected.

## 2. Experimental Section

### 2.1. Animals

Male mice aged between 15–18 weeks with differently expressed PGC-1α were used. This included wild-type mice (WT), mice with a selective ablation of PGC-1α in skeletal muscle (KO), and mice overexpressing PGC-1α in skeletal muscle (OE). Mice breeding pairs were kindly provided by Prof. Handschin (University of Basel, Switzerland). Knockout (KO) mice line had been created using the Cre/LoxP system with floxed“ *Ppargc1α*, as previously described [25]. Tissue specificity was obtained with Cre recombinase under myogenin and MEF2C promoter sequences [26]. Mice overexpressing PGC-1α were generated using the DNA microinjection technique [27]. Wild-type (WT) mice consisted of male mice from both KO and OE mouse lines without gene mutations.

Mice were housed in neutral temperature environment (22 ± 2 °C) on a 12-h dark or light cycle. Access to food and water was *ad libitum*. All experiments were performed in agreement with the guidelines from Directive 2010/63/EU of the European Parliament on the protection of animals used for scientific purposes. Experiments were reviewed and accepted by the cantonal veterinary authority of Basel (License number 2847 approved on 21 July 2016), and were performed in agreement with the guidelines for care and use of laboratory animals.

After one week of acclimatization, mice were randomly divided into 6 groups as follows: (1) WT animals control treated with water (Ctl, *n* = 14); (2) WT animals treated with simvastatin at 5 mg/kg per day (Simv, *n* = 14); (3) KO mice treated with water (Ctl, *n* = 14); (4) KO mice treated with simvastatin at 5 mg/kg per day (Simv, *n* = 14), (5) OE mice treated with water (Ctl, *n* = 14); and (6) OE mice treated with simvastatin at 5 mg/kg per day (Simv, *n* = 14). Mice were treated daily by oral gavage for three weeks. During this period, body weight, food, and water intake were measured daily to monitor the health status during the treatment period. The simvastatin dose was calculated as described by Reagen-Shaw et al. [28] and corresponds to approximately 0.4 mg/kg per day in humans, which is a normally-used dose. Moreover, this dose was selected based on our previous studies where mice showed peak plasma concentrations similar to human patients treated with 40 mg of simvastatin per day [14,21,29].

### 2.2. Intraperitoneal Glucose Tolerance Test (iGTT)

The iGTT was performed after 21 days of treatment according to standard operating procedures [30]. Mice (*n* = 10) were fasted for 6 h after which blood was drawn from the tail vein to measure basal blood fasting glucose with a glucometer (Bayer Contour, Bayer, Leverkusen, Germany) and basal insulin concentration from plasma as described below. After the basal glucose and insulin measurement, mice were injected intraperitoneally with 2 g/kg of glucose (40% glucose, B. Braun, Melsungen, Germany). Subsequently, glucose was measured in blood from the tail vein at 15, 30, 60, 90, and 120 min after the glucose injection. Additional blood was taken for insulin measurements at 15 and 30 min following the glucose injection. This blood (and the blood obtained before glucose injection) was centrifuged at 10,000 *g* for 5 min at 4 °C to obtain plasma. Afterwards, 10 µL plasma aliquots were used to determine insulin concentrations with a Mouse/Rat Insulin kit (MSD, Maryland, Rockville, MD, USA). Glucose AUC_0–120 min_ and insulin AUC_0–30 min_ were determined using the trapezoid method.

### 2.3. Sample Collection

After three weeks of treatment, mice were anesthetized with an intraperitoneally injection of ketamine (160 mg/kg, Ketasol, Graeub, Bern, Switzerland) and xylazine (20 mg/kg, Rompun, Bayer, Leverkusen, Germany). After having obtained deep anesthesia, blood was sampled from the heart into an EDTA-coated tube and centrifuged at 3000 rpm for 15 min at 4 °C to obtain plasma. Gastrocnemius and quadriceps muscles were dissected, and immediately frozen in liquid nitrogen, to be stored at −80 °C for later analysis.

### 2.4. In Vivo Skeletal Muscle Glucose Uptake

The day before the sacrifice, mice (*n* = 4) were fasted overnight for 12 h to ensure low plasma insulin concentrations. Glucose uptake was stimulated by i.p. injection of a solution containing glucose and deoxyglucose in order to induce insulin release [31,32]. The intraperitoneally (10 mL/kg body weight) injected solution contained glucose (2 g/10 mL), 2-deoxyglucose (2-DG; 32.8 mg/10 mL), and 600 µCi/10 mL ^3^H-labeled 2-DG (^3^H-2-DG) (Anawa, Switzerland). Blood was drawn at 0, 15, and 30 min post-injection from the tail vein to measure ^3^H-2-DG present in the plasma (obtained from blood centrifuged at 10,000 *g* for 5 min at 4 °C). After the 30 min time point, mice were immediately sacrificed for the dissection of gastrocnemius muscles. To determine the ^3^H-2-DG activity, a plasma aliquot was mixed with 10 µL BaSO4 adsorbent solution containing 0.3 N Ba(OH)_2_ and 0.3 N ZnSO_4_ in a 1:1 ratio. This aliquot was centrifuged 16,000 *g* for 2 min at 4 °C and then the supernatant was used to measure ^3^H activity by scintillation counting. The plasma ^3^H-2-DG activity was used to determine AUC^3^_H-DG 0–30 min_ using the trapezoid method. To determine the accumulated ^3^H-2-DG in skeletal muscle, the phosphorylated form of ^3^H-2-DG (^3^H-2-DG-P_muscle_) was extracted from gastrocnemius muscle. Approximately 50 mg of muscle was homogenized with a mikrodismembrator for 1 min at 3000 rpm (Sartorius Stedim Biotech, Aubagne, France) and then neutralized with 0.5% perchloric acid. Homogenates were centrifuged at 4000 *g* for 20 min at 4 °C, the supernatants separated, and the pH adjusted to 7.5 ± 0.2 using a solution containing 6 M NaOH and 0.5 M HEPES. The supernatant was centrifuged again at 4000 *g* for 20 min at 4 °C and the resulting supernatant aliquoted (twice 100 µL) to measure the corresponding ^3^H-labeled activity by scintillation counting. To the first aliquot, 400 µL of the BaSO_4_ adsorbent solution mentioned above was added to remove ^3^H-2-DG-P, allowing the measurement of the unphosphorylated ^3^H-2-DG form only. The second aliquot was used for the total ^3^H activity (^3^H-2-DG and ^3^H-2-DG-P). The ^3^H-2-DG-P_muscle_ concentration inside the muscle were calculated as the difference between the two aliquots.

2-DG clearance (Cl_2-DG_) by skeletal muscle was calculated as described previously [33]:

(1) Cl2−DG=H3−2−DG−PmuscleAUC H3−2−DG0−30 min

Glucose transport into skeletal muscle (metabolic index, R_g_) was calculated as described previously [33]:

(2) $$R_{g}={\mathrm{Cl}}_{2-\mathrm{DG}}\times {\mathrm{glucose}}_{\mathrm{plasma}}$$

Plasma glucose and plasma insulin concentrations and the corresponding AUCs were determined as described for the iGTT in Section 2.2.

### 2.5. Quantification of Insulin Sensitivity

The quantitative insulin sensitivity check index (QUICKI) was calculated based on a previous publication [34]:

(3) $$\mathrm{QUICKI}=\frac{1}{\left[\log\;\left(\mathrm{fasting}\;\mathrm{insulin}\right)\;+\;\log\;\left(\mathrm{fasting}\;\mathrm{glucose}\right)\right]}$$

The insulin sensitivity index by Matsuda (ISI_Matsuda_) was calculated as:

(4) $${\mathrm{ISI}}_{\mathrm{Matsuda}}=\frac{1000}{\sqrt{G_{0}\times I_{0}\times G_{\mathrm{mean}}\times I_{\mathrm{mean}}}}$$

where G_mean_ is the mean blood glucose concentration during the iGTT and I_mean_ the mean plasma insulin concentration during the iGTT [35].

In addition, we estimated insulin sensitivity of the skeletal muscle (ISI_muscle_) by dividing the skeletal muscle glucose uptake (R_g_) by the average insulin concentration:

(5) $${\mathrm{ISI}}_{\mathrm{muscle}}=\frac{R_{g}}{{\mathrm{AUC}}_{\mathrm{insulin}\;0-30\;\min}}\times 30\frac{µ\mathrm{mol}\;\mathrm{glucose}\;\mathrm{per}\;100\;g\;\mathrm{muscle}}{\mathrm{ng}\;\mathrm{insulin}\;\mathrm{per}\;\mathrm{mL}\;\mathrm{plasma}}$$

This approach is based on the publication of Soonthornpun et al. [36] who indirectly calculated the peripheral glucose uptake after an oral glucose load and showed an excellent correlation with insulin sensitivity obtained with a glucose clamp.

### 2.6. Muscle Glycogen Content

The muscle glycogen content was measured in gastrocnemius muscle extracts according to Harris [37]. Homogenized tissue samples (20 mg) were lysed in alkaline solution (1 mg tissue per 30 μL 0.1 M NaOH) and incubated at 80 °C for 20 min under constant shaking. The supernatant of the homogenate was then neutralized with a solution containing 0.15 M citric acid and 0.15 Na_2_HPO_4_, pH 3. The glycogen was hydrolyzed with 10 μL of amyloglucosidase (10 mg/mL). After conversion to glucose, a 50 μL aliquot was loaded into a 96 well plate. The reaction mix (containing 0.04 M Mg-acetate tetrahydrate, 0.14 M triethanolamine hydrochloride, 1.37 mM EDTA, 1.14 mM ATP, 1.38 mM dithiothreitol, 1.44 mM NAD lithium salt, 12.7 U/mL glucose-6-phosphate dehydrogenase) was added to each well and background sample absorbance was measured at 366 nm after 5 min at room temperature (RT). To start the reaction, 1500 U/mL of hexokinase was added. The plate was incubated at room temperature for 15 min and the absorbance was measured at 366 nm. Measured values were corrected with background sample absorbance and calculated based on a standard curve with known amounts of glucose. Results were normalized to tissue content and are expressed as μmol/g tissue.

### 2.7. Quantitative Real-Time PCR

Total RNA was obtained from gastrocnemius (40 mg) using the RNeasy Fibrous Tissue Mini Kit (QIAGEN GmbH, Hilden, Germany) according to the manufacturer’s instructions. RNA was stored at −80 °C until the reverse transcription reaction was performed. cDNA was synthetized from 0.5 µg total RNA with the Omniscript RT kit (QIAGEN GmbH, Hilden, Germany). To perform the real-time PCR reaction, cDNA was mixed with each primer (sense and antisense at 0.3 µM final concentration), SYBR Green (Roche Diagnostics, Mannheim, Germany) as a fluorescent dye and H2O. The real-time PCR measurement of individual cDNAs was performed in triplicate using SYBR Green dye to measure duplex DNA formation with the ViiA™ 7 Real-Time PCR System (Applied Biosystems, Waltham, MA, USA). The primers sequences were designed using information contained in the public database GenBank of the National Center for Biotechnology Information (NCBI). The sequences of the primer sets used are: *Glut4* forward primer 5′-GATTCTGCTGCCCTTCTGTC-3′; *Glut4* reverse primer 5′-TGGACGCTCTCTCTCCAACT-3′; *Hk2* forward primer 5′-CCCTGCCACCAGACGAAA-3′; *Hk2* reverse primer 5′-GACTTGAACCCCTTAGTCCATGA-3′; *Ppargc1a* forward primer 5′- AATGCAGCGGTCTTAGCACT-3′; *Ppargc1a* reverse primer 5′- ACGTCTTTGTGGCTTTTGCT-3′, *18s* forward primer 5′-AGTCCCTGCCCTTTGTACACA-3′; and *18s* reverse primer 5′-CGATCCGAGGGCCTCACTA-3′. Quantification of gene expression was performed as described previously [38], using the *18s* gene as the housekeeping gene.

### 2.8. Western Blots

Muscle samples (30 mg) from quadriceps were first pulverized with a mikrodismembrator for one min at 3000 rpm (Sartorius Stedim Biotech, Aubagne, France) and lysed on ice in PhosphoSafe™ Extraction Reagent (Merck, Darmstadt, Germany), and then centrifuged at 16,000 *g* at 4 °C for 10 min. The protein content in the supernatant was determined with a Pierce BCA protein assay kit (ThermoFisher Scientific, Waltham, MA, USA). Fifty micrograms of protein was loaded onto the NuPAGE 4–12% Bis-Tris gel (Life technologies, Rockville, MD, USA). Gels were run at 140 V and then, after separation, electroblotted to nitrocellulose membranes (Bio-Rad, Hercules, CA, USA). Proteins were immunodetected using antibodies against Glut4 (07-1404 1:1000, Merck, Darmstadt, Germany), hexokinase (ab209847, 1:1000, Abcam, Cambridge, UK), and eEF2 (#2332, 1:1000, Cell Signaling Technology, Danvers, USA). Membranes were then probed with secondary HRP conjugated antibodies against rabbit (sc-2357, 1:2000, Santa Cruz Biotechnology, Dallas, USA) or mouse (sc-516102, 1:2000, Santa Cruz Biotechnology) after which a chemiluminescent substrate (Clarity Western ECL substrate; Bio-Rad Laboratories, Hercules, CA, USA) was added to visualize the bands. Protein expression was quantified using the Fusion Pulse TS device (Vilber Lourmat, Oberschwaben, Germany).

### 2.9. Statistical Analysis

Data are presented as means ± SEM. Statistical analyses were performed using 2-way ANOVA followed by False Discovery rate (FDR) correction for multiple comparisons (Two-stage step-up method of Benjamini, Krieger and Yekutieli) to localize differences. Analyses were performed using the GraphPad Prism 8 (Graph Pad Software, Inc., San Diego, CA, USA). Significance was set at *p* < 0.05.

## 3. Results

### 3.1. Physiological Characterization of the Mice

**Figure 1:**
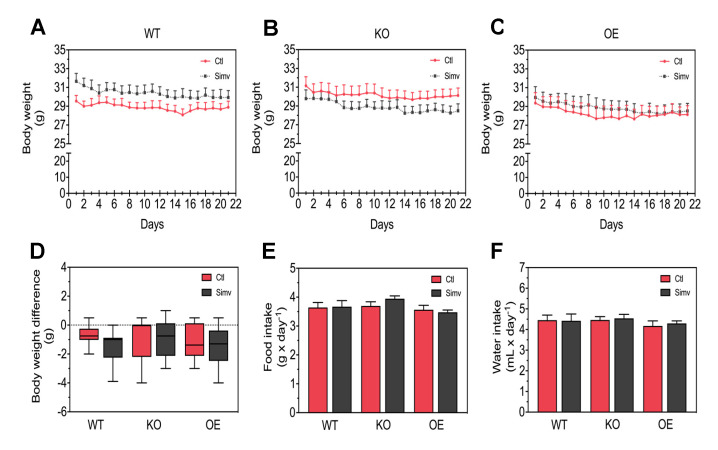
General physiological parameters. Physiological parameters, such as body weight, water and food intake, were monitored daily during the treatment. Body weight is shown as the average during the three weeks of treatment for WT mice (**A**), KO mice (**B**), and for OE mice (**C**). The change in body weight is shown as a difference in grams between the body weight at the end and at the start of treatment (**D**). Food (**E**) and water (**F**) intake are presented as an average value during the treatment. Data are presented as mean ± SEM of 10 animals per group. There were no statistically significant differences between the groups and for treatment with simvastatin. Ctl, control; KO, PGC-α knock-out mice; OE, PGC-1α overexpressing mice; Simv, simvastatin; WT, wild-type.

In order to validate our mouse models, we measured first the mRNA expression levels of *Ppargc1a* in gastrocnemius muscles from WT, KO, and OE mice. As expected, we found that the mRNA expression of *Ppargc1a* was significantly decreased in KO mice and increased in OE mice when compared to WT mice (Supplementary Figure S1). Mice were assessed daily for their health status by monitoring their body weight over the treatment of three weeks with either simvastatin or water. Simvastatin (5 mg/kg per day) did not affect body weight and there were no differences between the mouse models (Figure 1A–C). To better characterize the change in body weight, we plotted the difference between day 1 and day 21 (Figure 1D). In this presentation, there were no differences between the mouse models investigated, but simvastatin decreased the body weight of WT mice over the treatment period. Moreover, neither PGC-1α expression nor simvastatin affected the mean daily intake of food or water (Figure 1E,F). Accordingly, the daily food and water intake was stable and not affected by PGC-1α or simvastatin over the course of the treatment (Supplementary Figure S2). These data indicate that this dose of simvastatin was well-tolerated independently of PGC-1α expression.

### 3.2. Simvastatin Impaired Glucose Homeostasis in WT and OE Mice Whereas KO Mice Showed Already Higher Blood Glucose Concentrations during the iGTT Without Simvastatin Treatment

**Figure 2:**
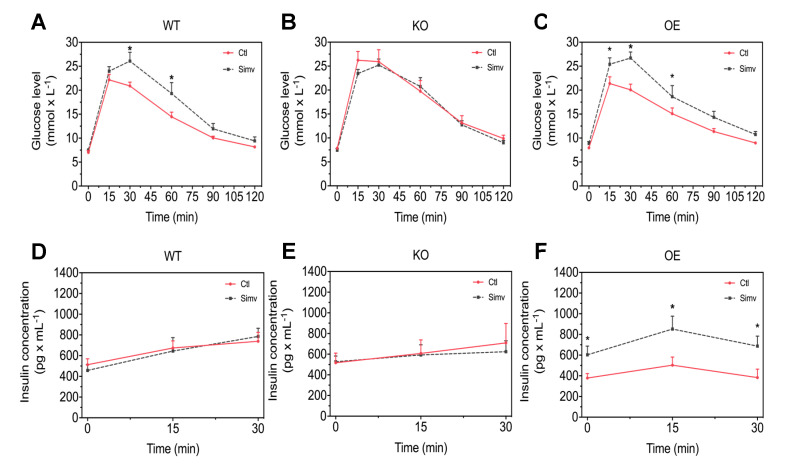
Intraperitoneal glucose tolerance test. Mice were studied after starvation for 6 h. Blood glucose concentrations were measured over 120 min and insulin plasma concentrations over 30 min after intraperitoneal injection of 2 g/kg glucose. Glucose concentrations are shown for WT mice (**A**), KO mice (**B**), and for OE mice (**C**) and plasma insulin concentrations for WT mice (**D**), KO mice (**E**) and for OE mice (**F**). After two-way ANOVA analysis, treatment factor was significant in (**A**,**C**,**F**). Data are presented as mean ± SEM of 10 animals per group. * *p* < 0.05 between simvastatin-treated and respective control mice. Ctl, control; KO, PGC-1α knock-out mice; OE, PGC-1α overexpressing mice; Simv, simvastatin; WT, wild-type.

As a next step, we investigated whether PGC-1α expression and/or treatment with simvastatin might affect glucose homeostasis. For that, we performed an intraperitoneal GTT to compare blood glucose and insulin concentration changes over time in WT, KO, and OE mice treated or not with simvastatin in mice starved for 6 h. The glucose and insulin concentrations before and at different time points after the glucose load are shown in Figure 2 and in the Supplementary Table S1. The glucose concentrations before the blood glucose load were in a range of 7 to 9 mM and were neither affected by the expression of PGC-1α, nor by simvastatin treatment (Figure 2A–C). In WT and KO mice, the basal plasma insulin concentrations were approximately 500 pg/mL and were not affected by simvastatin (Figure 2D,E). In comparison, in OE mice treated with water, the basal insulin concentration was numerically lower (379 pg/mL) but higher in OE mice treated with simvastatin (603 pg/mL) (Figure 2F). After the glucose load, WT mice treated with simvastatin showed higher blood glucose concentrations at 30 and 60 min compared to the water-treated WT mice (Figure 2A). The plasma insulin concentrations increased linearly after the glucose load to approximately 750 pg/mL at 30 min and were not different between simvastatin-treated and water-treated WT mice (Figure 2D). KO mice treated with water showed overall higher glucose concentrations as compared to water-treated WT mice (Figure 2A,B, Supplementary Table S1) and treatment with simvastatin did not significantly affect blood glucose concentrations (Figure 2B). The plasma insulin concentrations increased linearly after the glucose load up to approximately 600 pg/mL (Figure 2E). This increase was not significantly affected by simvastatin and slightly less than in WT mice. Water-treated OE mice showed the expected increase in the blood glucose concentrations after the glucose load, which was similar to water-treated WT mice (Figure 2C and Supplementray Table S1). The increase in the plasma insulin concentration was less accentuated (from approximately 400 to 430 pg/mL) than in WT or KO mice and reached its maximal value already at 15 min (Figure 2F). Similar to WT mice, treatment of OE mice with simvastatin was associated with significantly higher blood glucose concentrations compared to water-treated OE mice 15, 30, and 60 min after the glucose load (Figure 2C). The plasma insulin concentrations were already significantly higher in simvastatin-treated compared to water-treated OE mice under basal conditions and proceeded parallelly to water-treated OE mice after the glucose load with a maximal value at 15 min (Figure 2F).

**Figure 3:**
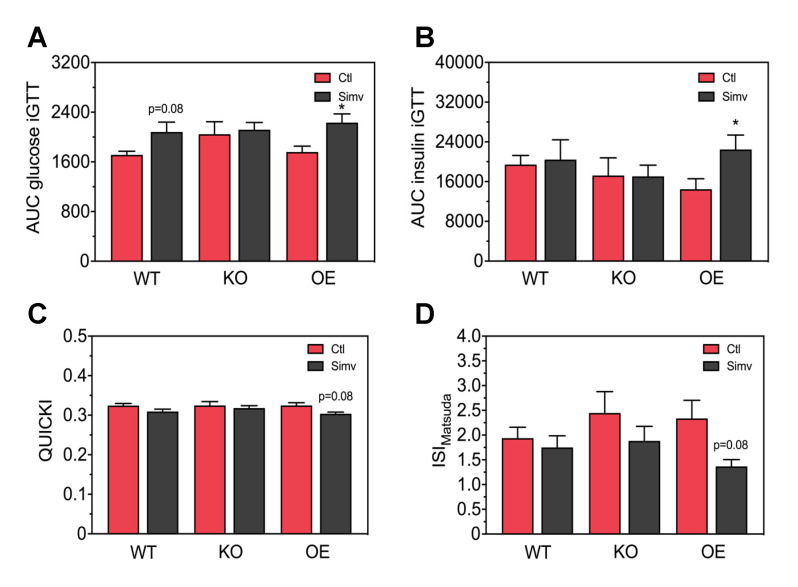
Area under the curves for the glucose blood and insulin plasma concentrations during the intraperitoneal glucose tolerance test, and insulin sensitivity indexes. Blood glucose concentrations (**A**) and insulin plasma concentrations (**B**), measured during the glucose tolerance test, were quantified as the area under the curve using the trapezoid method. Surrogate indexes for insulin sensitivity were calculated as the QUICKI (**C**) and the insulin sensitivity index by Matsuda (**D**). After two-way ANOVA analysis, the treatment factor was significant in (**A**,**B**,**D**) and there was no significant effect of the animal models and no significant interaction. Data are presented as mean ± SEM of 10 animals per group. * *p* < 0.05 between simvastatin-treated and respective control mice. Ctl, control; KO, PGC-1α knock-out mice; OE, PGC-1α overexpressing mice; Simv, simvastatin; WT, wild-type.

To better quantify the results acquired from the iGTT, we calculated the area under the curve (AUC) for the blood glucose and for the plasma insulin concentration. The AUC_glucose 0–120 min_ was not significantly affected by PGC-1α expression in both water-treated and simvastatin-treated mice (Figure 3A). Treatment with simvastatin was associated with a numerical increase in the AUC_glucose 0–120 min_ in WT mice (*p* = 0.08) and a significant increase in AUC_glucose 0–120 min_ in OE mice. As shown in Figure 3B, PGC-1α expression had no significant impact on the AUC_insulin 0–30 min_ in water-treated mice. Treatment with simvastatin had no effect in WT and KO mice, but increassed the AUC_insulin 0–30 min_ significantly in OE mice.

Next, we assessed glucose homeostasis by calculating the quantitative insulin sensitivity check index (QUICKI) (Figure 3C). In water-treated mice, the QUICKI was not affected by PGC-1α expression. Simvastatin decreased the QUICKI numerically in OE mice (*p* = 0.08). In addition, we calculated the insulin sensitivity index according to Matsuda (ISI_Matsuda_), which describes whole-body insulin sensitivity taking also the iGTT data into account [35]. As shown in Figure 3D, the ISI_Matsuda_ was not significantly affected by PGC-1α expression in both water-treated mice. Treatment with simvastatin showed a trend to decrease the ISI_Matsuda_ in all mouse models investigated, whereby the decrease in OE mice was close to significance (*p* = 0.08).

### 3.3. Simvastatin Impaired Glucose Uptake and Glycogen Muscle Reserves in WT and KO Mice, But Not in OE Mice

**Figure 4:**
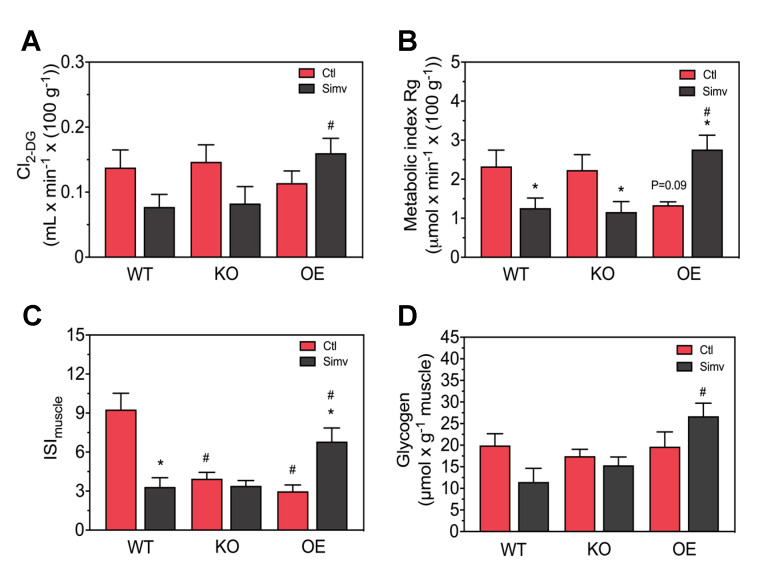
Glucose uptake and muscle glycogen. Muscle specific glucose uptake is shown as clearance for 2-deoxyglucose (2-DG) Cl_2-DG_ (**A**) and as the metabolic index (Rg), which reflects skeletal muscle glucose uptake (**B**). The ratio of muscle glucose uptake and the average insulin blood concentration was calculated as a muscle-specific marker of insulin sensitivity (ISI_muscle_) (**C**). Glycogen content was measured from gastrocnemius muscle (**D**). After two-way ANOVA analysis, we found significant treatment effects in (**B**,**C**), significant animal model effects in (**A**–**D**) and the interaction between treatment and animal model was significant in (**A**–**C**). Data are presented as mean ± SEM of 4–8 animals per group. * *p* < 0.05 between simvastatin-treated and respective control mice and # *p* < 0.05 between KO or OE and WT mice within the same treatment group. Ctl, control; KO, PGC-1α knock-out mice; OE, PGC-1α overexpressing mice; Simv, simvastatin; WT, wild-type.

Since the iGTT and the ISI_Matsuda_ describe the whole body glucose homeostasis and are not specific for skeletal muscle, we assessed directly the in vivo transport of glucose into skeletal muscle in order to obtain muscle-specific data in mice starved for 12 h. As shown in Figure 4A, PGC-1α expression had no significant effect on 2-DG clearance by skeletal muscle in water-treated mice. Treatment with simvastatin showed a trend to decrease skeletal muscle 2-DG clearance in WT and KO mice and a trend to increase 2-DG clearance in OE mice (Figure 4A). In simvastatin-treated mice, the 2-DG clearance were significantly higher in OE compared to WT mice.

Multiplication of the 2-DG clearance with the average plasma glucose concentration yields glucose transport into skeletal muscle, which is shown in Figure 4B (the plasma glucose and plasma insulin concentrations obtained after 12 h of starvation are given in Supplementary Table S1). Again, PGC-1α expression did not significantly influence skeletal muscle glucose transport in water-treated mice. Simvastatin significantly decreased the skeletal muscle glucose transport in WT and KO mice, but increased this transport in OE mice (Figure 4B). In simvastatin-treated mice, glucose transport was significantly higher in OE compared to WT and KO mice.

Division of glucose transport by the average insulin concentration yields a marker for skeletal muscle insulin sensitivity that we named ISI_muscle_. ISI_muscle_ describes the ability of the muscle to take up glucose depending on the actual insulin concentration. As shown in Figure 4C, ISI_muscle_ in water-treated mice was significantly decreased in KO and OE mice compared to WT mice. In WT mice, simvastatin decreased ISI_muscle_ significantly, whereas simvastatin did not further decrease ISI_muscle_ in KO mice (Figure 4C). In contrast, in OE mice simvastatin significantly increased the ISI_muscle_.

Since glucose uptake was impaired by simvastatin in WT and KO mice and increased in OE mice, we checked whether skeletal muscle glycogen stores were also affected. As shown in Figure 4D, the skeletal muscle glycogen content was not affected by PGC-1α expression in water-treated mice. Treatment with simvastatin decreased the skeletal muscle glycogen stores in WT and increased the glycogen stores in OE mice by trend (Figure 4D). In simvastatin-treated mice, the skeletal muscle glycogen stores were significantly higher in OE compared to WT and KO mice.

### 3.4. Simvastatin Had No Significant Impact on Skeletal Muscle GLUT4 and Hexokinase Protein Expression in WT and KO Mice But Increased the Expression of Both Proteins in OE Mice

**Figure 5:**
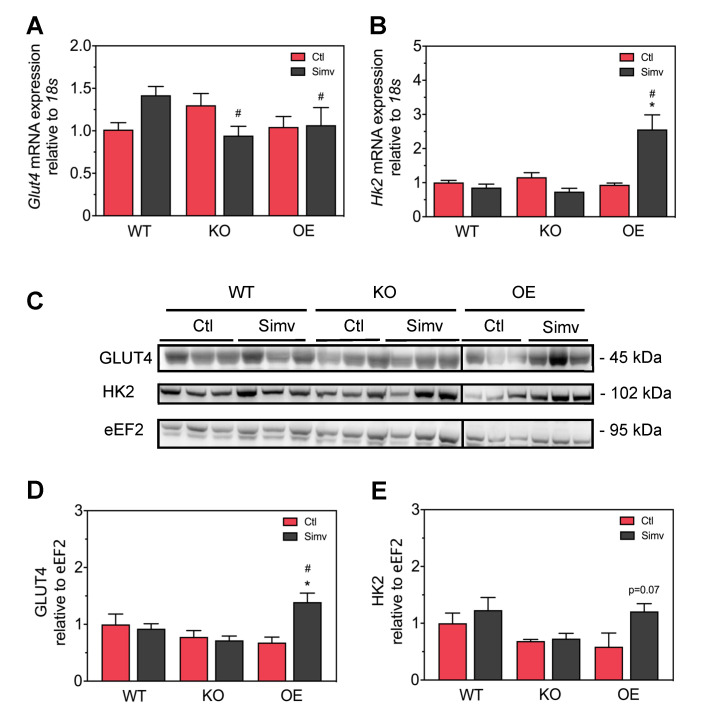
mRNA and protein expression of Glut4 and hexokinase. Intensity of the protein bands acquired from quadriceps muscle homogenates was quantified by normalizing them to eEF2 expression. The mRNA expression of *Glut4* (**A**) and *Hk2* (**B**). Western blots showing protein expression of Glut4, HK2, and eEF2 (**C**). Quantification of Glut4 (**D**) and HK2 (**E**) protein expression normalized to eEF2. After two-way ANOVA analysis, the treatment factor was significant in (**B**,**D**), the animal model factor significant in (**A**,**B**,**D**) and the interaction between treatment and animal model significant in (**A**,**B**,**D**). Data are presented as mean ± SEM of 3–7 randomly chosen animals per group. * *p* < 0.05 between simvastatin-treated and respective control mice and # *p* < 0.05 between KO or OE and WT mice within the same treatment group. Ctl, control; KO, PGC-1α knock-out mice; OE, PGC-1α overexpressing mice; Simv, simvastatin; WT, wild-type.

To find a possible explanation for the effect of simvastatin on skeletal muscle glucose transport, we measured the mRNA and protein expression of Glut4 and Hk2 in skeletal muscle. As shown in Figure 5A, PGC-1α expression did not significantly affect *Glut4* mRNA expression in water-treated mice. Simvastatin increased the mRNA *Glut4* expression numerically in WT but decreased it in KO mice, while there was no significant effect in OE mice. Similarly, PGC-1α expression did not significantly affect *Hk2* mRNA expression in water-treated mice (Figure 5B). Simvastatin treatment did not affect *Hk2* mRNA in WT and KO mice, but increased *Hk2* mRNA expression significantly in OE mice. Regarding protein expression, PGC-1α expression did not affect Glut4 protein expression in water-treated mice (Figure 5C,D). Treatment with simvastatin did not significantly affect Glut4 protein expression in WT and KO mice, but increased it significantly in OE mice compared to water-treated OE mice. Similarly, PGC-1α expression did not significantly affect hexokinase protein expression in water-treated mice (Figure 5C,E). Treatment with simvastatin did not affect hexokinase protein expression in WT and KO mice but increased it by trend in OE mice (*p* = 0.07).

## 4. Discussion

The current study shows that both skeletal muscle PGC-1α expression and treatment with simvastatin affect whole body and skeletal muscle glucose metabolism and that the effect of simvastatin on the peak glucose concentrations in the iGTT, skeletal muscle glucose uptake and mRNA/protein expression of Glut4 and hexokinase depends on the skeletal muscle expression of PGC-1α.

The findings in WT mice in the current study confirm our previous observations regarding the effect of simvastatin on glucose homeostasis [21]. During the iGTT, simvastatin-treated mice developed higher glucose peak concentrations, while the insulin concentrations remained in the same concentration range than in water-treated mice. This is in line with the study of Larsen et al., which was performed in patients with dyslipidemia. These authors showed that during the oral glucose tolerance test (OGTT), blood glucose concentrations were significantly higher in patients treated with simvastatin compared to controls, while insulin plasma concentrations were similar in the two groups, a condition compatible with impaired insulin sensitivity [39]. They also found reduced mitochondrial uncoupling protein UCP3 content in skeletal muscle from the simvastatin-treated patients. UCP3 has been proposed to protect mitochondria and cells from accumulation of non-esterified fatty acids [40]. Accumulation of free fatty acids in muscle fibers has been proposed to be associated with skeletal muscle insulin resistance through the inhibition of the insulin signaling cascade [41]. Thus, the observation of glucose intolerance in mice and patients treated with simvastatin supports the view that statins decrease insulin sensitivity and thereby increase the risk for developing diabetes. As suggested by the decreased skeletal muscle glucose uptake and the reduced ISI_muscle_, simvastatin reduced skeletal muscle insulin sensitivity in WT mice, which is compatible with the above-mentioned mechanism and our previous study in C2C12 cells [42].

The effects of PGC-1α knock-out on glucose homeostasis have been described in detail by Handschin et al. [25]. Skeletal muscle-specific PGC-1α knock-out is associated with impaired pancreatic insulin secretion, which is due to increased production and secretion of IL-6 from skeletal muscle that damages the architecture of Langerhans islets and the function of β-cells [25]. The data in water-treated KO mice in the current study are in line with these findings. Under basal conditions, blood glucose and plasma insulin concentrations were comparable, but during the iGTT the blood glucose concentrations were higher in KO mice than in WT mice whereas the insulin plasma concentrations remained in the same range. In contrast to WT mice, simvastatin did not further deteriorate glucose homeostasis in KO mice as judged from the iGTT. However, simvastatin significantly reduced skeletal muscle glucose uptake in KO mice. This decrease may be explained by impaired mRNA and protein expression of Glut4, which can be rate-limiting for skeletal muscle glucose uptake [33]. Since simvastatin did not further deteriorate whole body glucose homeostasis in KO mice, we can conclude that the negative effect of PGC-1α knock-out on insulin secretion is more important for glucose homeostasis in KO mice than the inhibition of skeletal muscle glucose uptake by simvastatin.

The effect of PGC-1α overexpression on glucose homeostasis is controversial. Choi et al. reported a decrease in skeletal muscle insulin sensitivity particularly in mice fed a high fat diet, which was attributed to accumulation of fatty acid metabolites inhibiting insulin signaling [43]. On the other hand, Summermatter et al. have shown that skeletal muscle insulin sensitivity can be maintained in mice with PGC-1α overexpression on a high fat diet when mice perform regular exercise [44]. The mice in the current study were fed a regular diet and kept without regular physical training. During the iGTT in OE mice treated with water, the AUC_glucose_ was not different from WT mice and the AUC_insulin_ was numerically lower. On the other hand, the skeletal muscle glucose uptake of OE mice was reduced by approximately 50% compared to WT mice, mostly due to a low plasma glucose concentration. While the results of the iGTT suggested an at least maintained insulin sensitivity compared to WT mice, the decrease in the skeletal muscle glucose uptake was compatible with impaired skeletal muscle insulin sensitivity. A decrease in skeletal muscle insulin sensitivity is further suggested by the decrease in the ISI_muscle_ and by the low expression of Glut4 and hexokinase in skeletal muscle of OE mice. Based on these considerations, we can conclude that skeletal muscle insulin sensitivity was impaired in water-treated OE mice whereas systemic insulin sensitivity was maintained.

In comparison to water, treatment with simvastatin increased AUC_glucose_ and AUC_insulin_ during the iGTT, which was associated with a decrease in the QUICKI and the ISI_Matsuda_, indicating impaired systemic insulin sensitivity. These findings indicate that OE mice, in contrary to our hypothesis, are at least as sensitive as WT mice to simvastatin and that, at 6 h of starvation, high plasma insulin concentrations are necessary to keep the blood glucose concentration in an acceptable range. In apparent discrepancy, simvastatin caused an increase in skeletal muscle glucose uptake, which was mainly due to an increase in muscle 2-DG clearance. Increased skeletal muscle glucose uptake by simvastatin can be explained by the observed increase in the protein expression of Glut4 and hexokinase, which can both be rate-limiting for skeletal muscle glucose uptake [33]. Since the ISI_Matsuda_ reflects whole body glucose metabolism, the discrepancy with the skeletal muscle glucose uptake can also be explained by impaired insulin sensitivity of non-muscular tissues, as for instance the liver, where orally ingested statins reach high concentrations. Furthermore, since we determined only glucose uptake by the gastrocnemius muscle, we cannot exclude the possibility that other muscles would yield a different result. The stimulation of Glut4 and hexokinase protein expression by simvastatin in OE mice, which appears to be the major cause of the increased skeletal muscle glucose uptake, cannot be explained by the currently available data. It is possible that it represents a reaction to compensate the negative effects of simvastatin on glucose homeostasis in OE mice. We plan to investigate this finding in future studies.

It is evident that the markers of insulin sensitivity used in this study produced different results, particularly in OE mice treated with simvastatin. Markers using the glucose and insulin concentrations before and/or during the iGTT such as the QUICKI and the ISI_Matsuda_ suggested impaired insulin sensitivity, whereas markers directly assessing skeletal muscle glucose homeostasis such muscle 2-DG clearance, muscle glucose uptake or ISI_muscle_ indicated improved muscle insulin sensitivity in simvastatin-treated OE mice. In diabetic patients, an insulin sensitivity index based on glucose consumption showed a much better correlation with insulin sensitivity determined by a glucose clamp study [36]. This suggests that markers assessing tissue-specific insulin sensitivity are preferable to general markers if insulin-sensitivity of a specific tissue is of interest.

In conclusion, simvastatin impaired skeletal muscle insulin sensitivity in WT mice, while KO mice exhibited impaired skeletal muscle insulin sensitivity already in the absence of simvastatin. In OE mice, simvastatin augmented muscular glucose uptake but impaired whole-body insulin sensitivity. The significant interaction between PGC-1α expression and simvastatin regarding peak blood glucose concentrations in the iGTT, skeletal muscle glucose uptake and muscle mRNA/protein expression of Glut4 and hexokinase indicates that simvastatin affected glucose homeostasis depending on PGC-1α expression.

## Supplementary Materials

The following are available online at https://www.mdpi.com/2227-9059/8/9/351/s1.
