# Supplementary material for: Simvastatin Impairs Glucose Homeostasis in Mice Depending on PGC-1α Skeletal Muscle Expression
Source: Biomedicines. 2020 Sep 15;8(9):351. doi: 10.3390/biomedicines8090351 (PMC7555587; doi:10.3390/biomedicines8090351)
Supplement: Supplementary file 1 [file biomedicines-08-00351-s001.zip › biomedicines-919087-supplementary_JB_Final.docx]

| **Table S1.** Basal glucose levels after 6 h of starvation (mM) | | | | | | |
| --- | --- | --- | --- | --- | --- | --- |
|  | WT Ctl | WT Simv | KO Ctl | KO Simv | OE Ctl | OE Simv |
| Mean | 7.0 | 7.5 | 7.9 | 7.4 | 8.0 | 8.9# |
| SEM | 0.4 | 0.3 | 0.3 | 0.4 | 0.2 | 0.4 |
| Basal insulin levels after 6 h of starvation (pg x mL^-1^) | | | | | | |
| Mean | 512 | 458 | 517 | 527 | 379 | 603 (*p*=0.07) |
| SEM | 57 | 56 | 93 | 55 | 42 | 84 |
|  |  |  |  |  |  |  |
| Glucose levels 30 min after glucose administration after 6 h of starvation (mM) | | | | | | |
|  | WT Ctl | WT Simv | KO Ctl | KO Simv | OE Ctl | OE Simv |
| Mean | 20.9 | 26.1* | 25.9# | 25.2 | 20.1 | 26.7* |
| SEM | 0.8 | 1.8 | 2.5 | 1.3 | 1.2 | 1.3 |
| Insulin levels 30 min after glucose administration after 6 h of starvation (pg x mL^-1^) | | | | | | |
| Mean | 739 | 786 | 710 | 624 | 383 (*p*=0.06) | 687 |
| SEM | 86 | 79 | 187 | 105 | 81 | 96 |
|  |  |  |  |  |  |  |
| Basal glucose levels after 12 h of starvation (mM) | | | | | | |
|  | WT Ctl | WT Simv | KO Ctl | KO Simv | OE Ctl | OE Simv |
| Mean | 4.9 | 4.9 | 5.0 | 5.3 | 4.9 | 6.9 |
| SEM | 0.7 | 0.6 | 0.7 | 1.7 | 0.5 | 0.2 |
| Basal insulin levels after 12 h of starvation (pg x mL^-1^) | | | | | | |
| Mean | 410 | 339 | 517 | 449 | 372 | 438 (*p*=0.06) |
| SEM | 126 | 135 | 128 | 234 | 100 | 97 |

**Blood glucose and plasma insulin levels after six or twelve hours of starvation**. Mice with differential PGC-1α expression were treated with either simvastatin or water and after three weeks of treatment, their basal glucose and insulin levels were measured after six or twelve hours of starvation. In mice starved for 6 hours, blood glucose and plasma insulin levels were also determined 30 minutes after intraperitoneal application of 2 g/kg glucose. Two-way ANOVA with False Discovery rate correction for multiple comparison revealed significant treatment factor and interaction for blood glucose levels 30 minutes after glucose administration (6 h starvation) and significant mouse model factor in control treated mice for basal glucose levels (6 h starvation). **p* < 0.05 between simvastatin‐treated and respective control mice and #*p* < 0.05 between KO or OE and WT mice within the same treatment group. Data are presented as mean ± SEM of 10 (starvation for 6 hours) or 4 (starvation for 12 hours) randomly chosen animals per group. KO, PGC‐1α knock‐out mice; OE, PGC‐1α overexpressing mice; WT, wild-type.


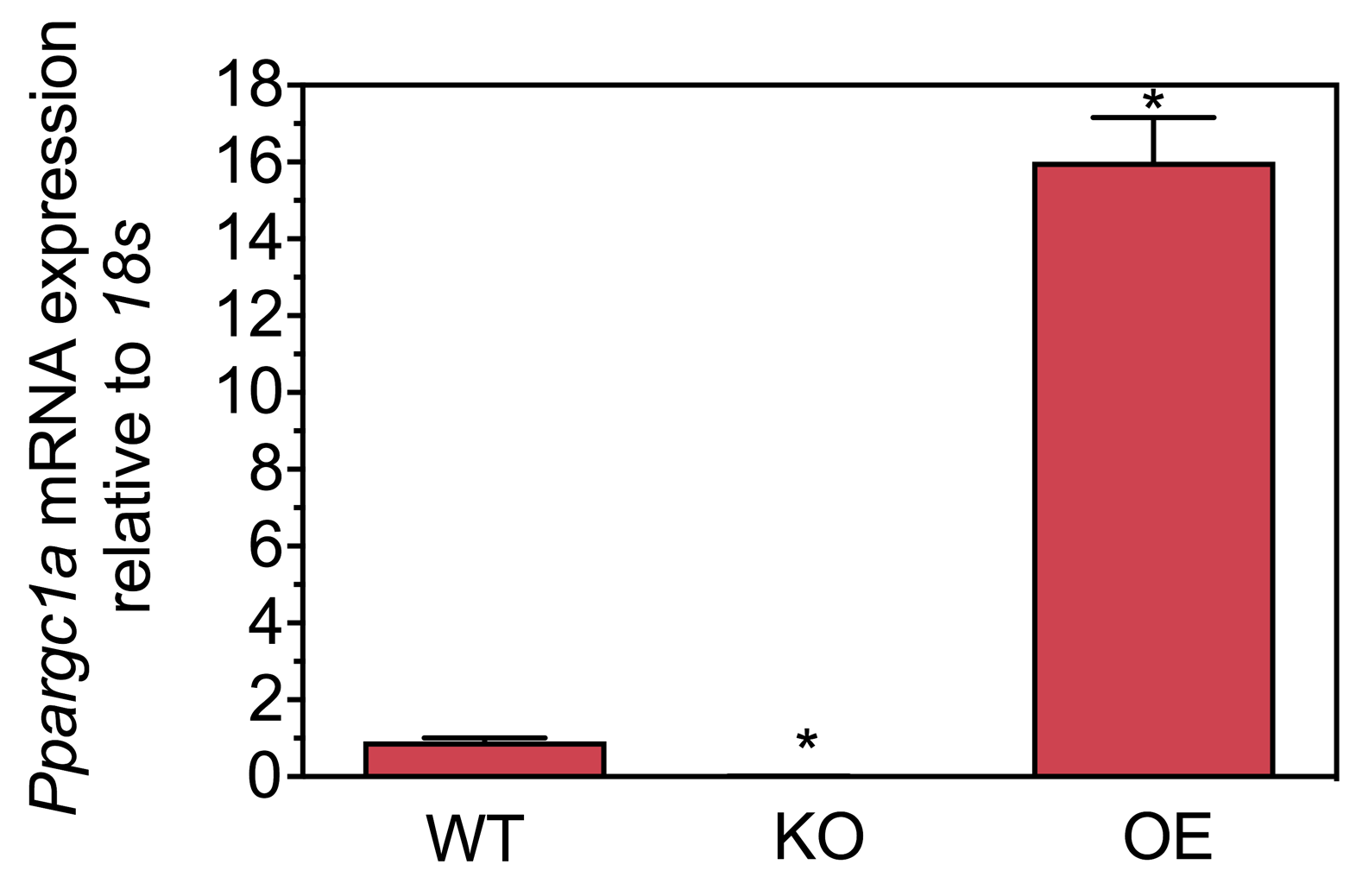


**Figure S1.** Expression of mRNA for Ppargc1a. Validation of mRNA Ppargc1a expression in mice gastrocnemius muscle extracts (A). After Brown-Forsythe and Welch ANOVA analysis and Dunnett`s multiple comparison, mouse model factor was significant. Data are presented as mean ± SEM of 7 randomly chosen animals per group. *P < 0.05 between KO or OE and WT mice. KO, PGC‐1α knock‐out mice; OE, PGC‐1α overexpressing mice; WT, wild-type.


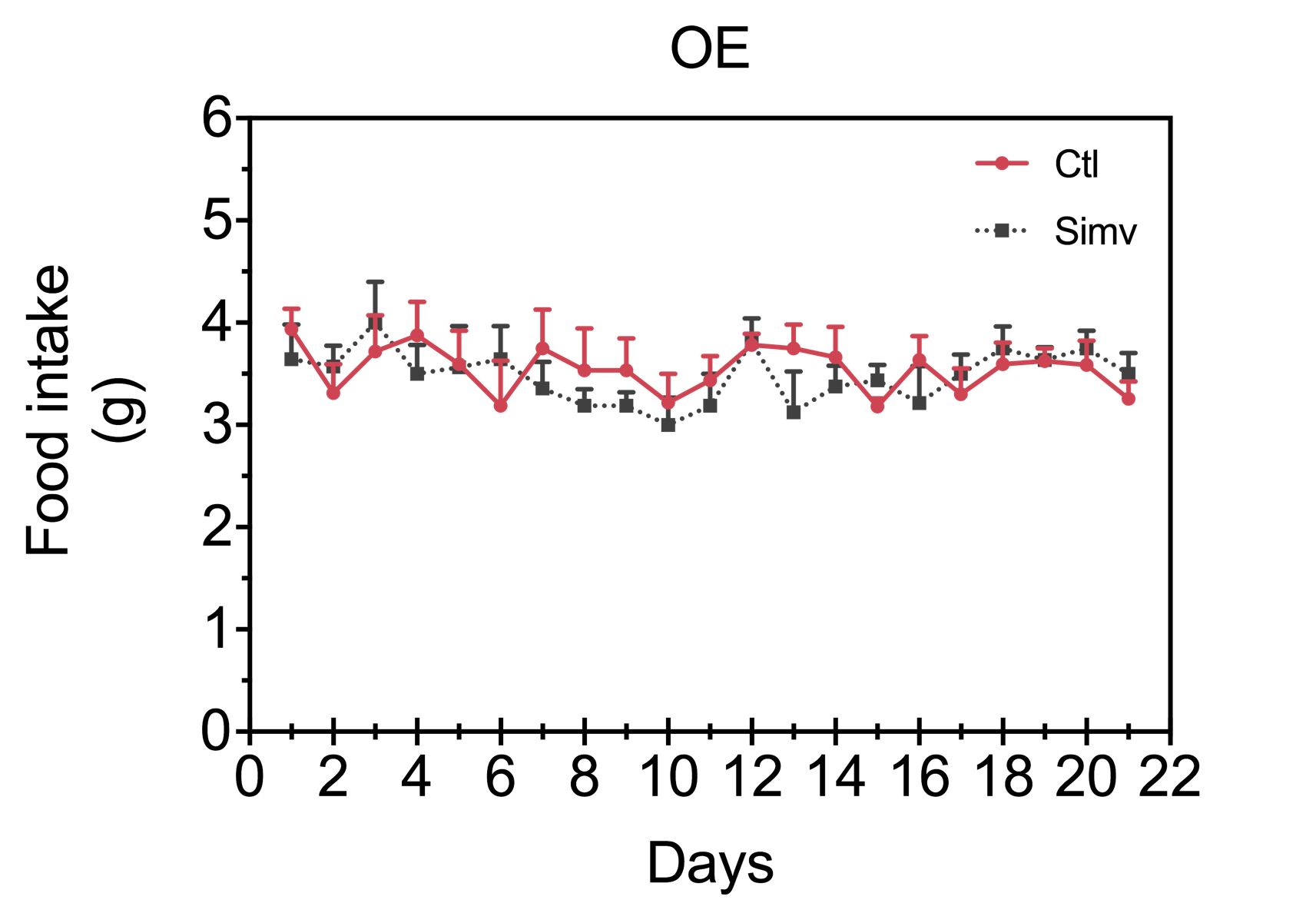

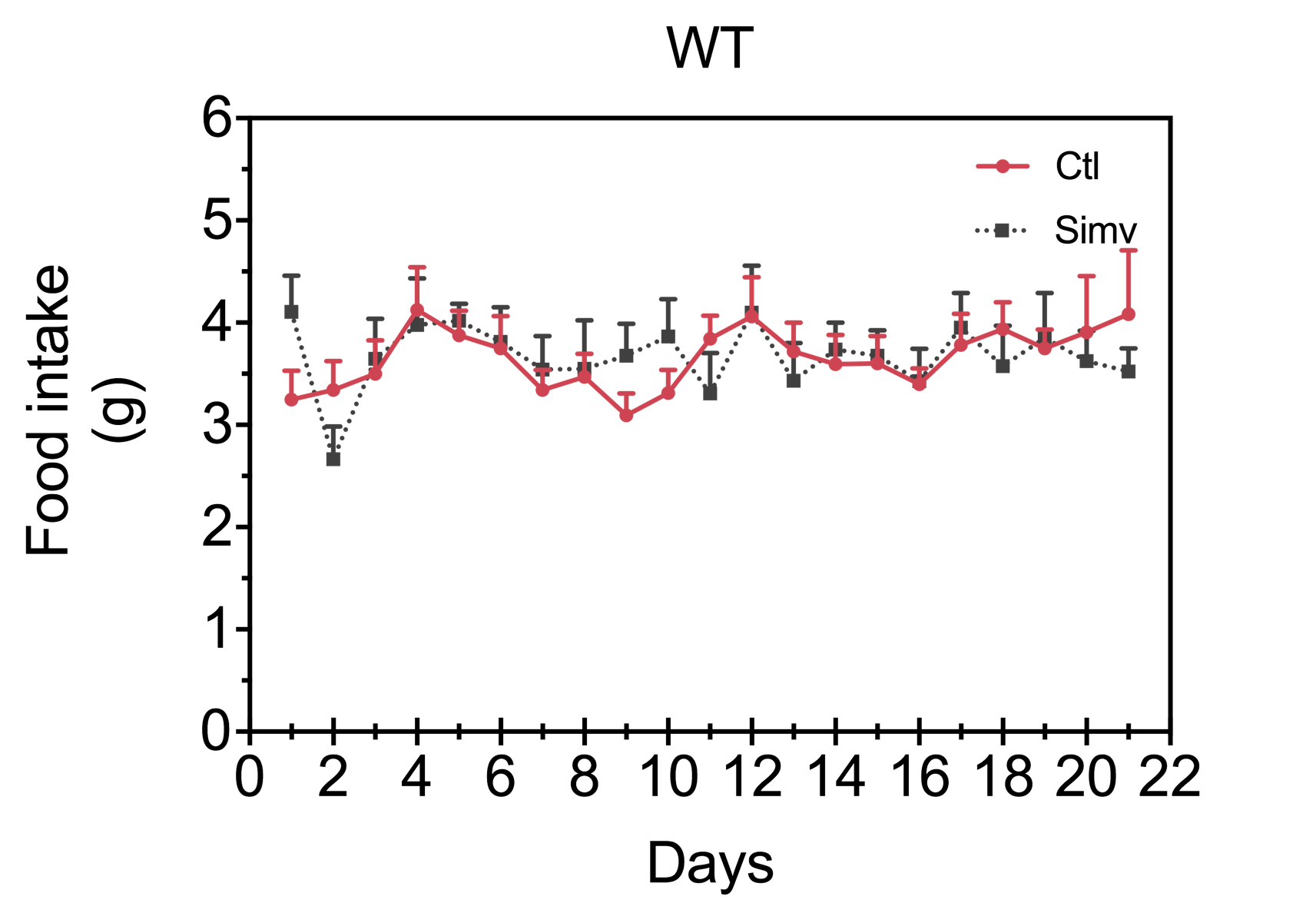

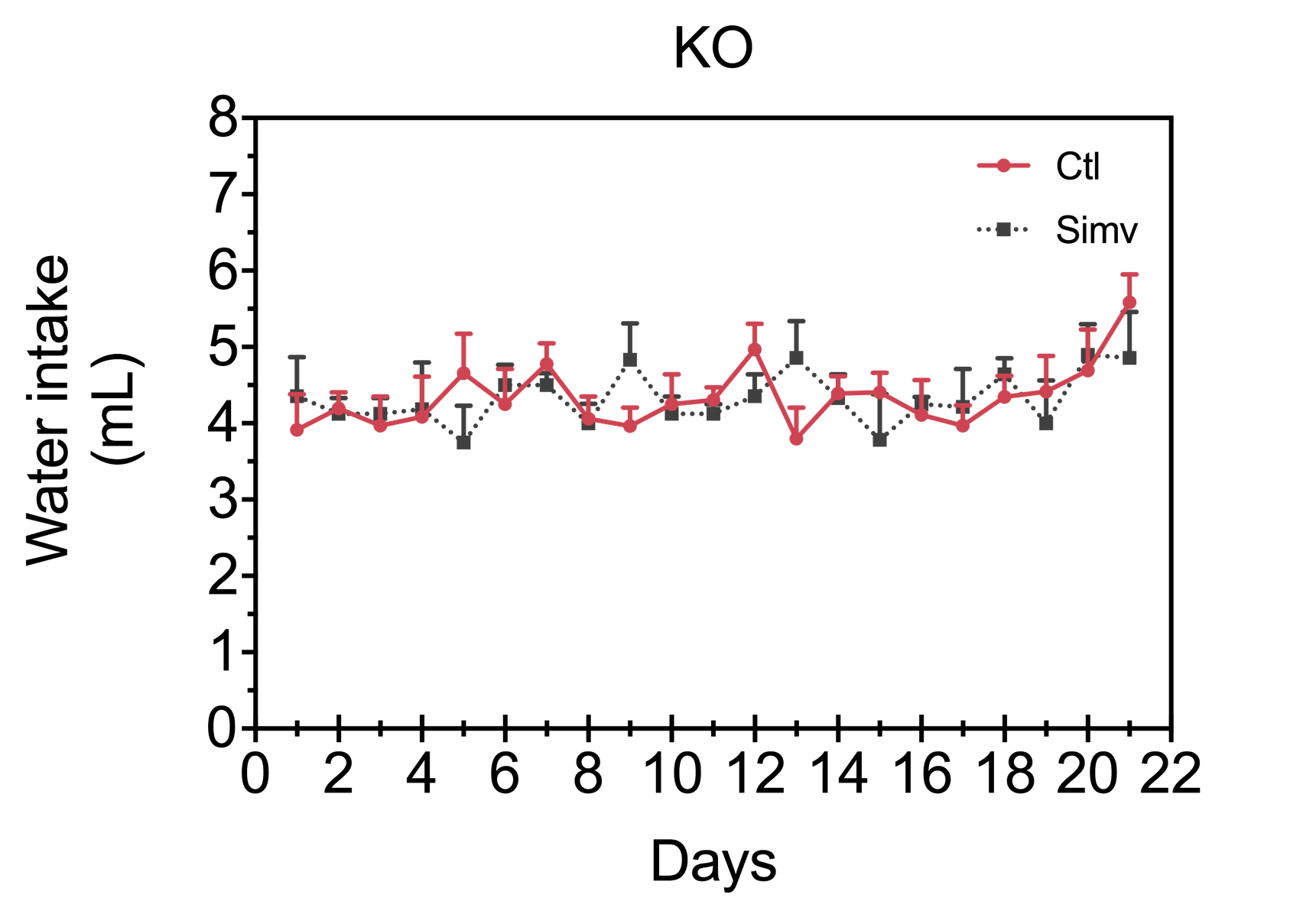

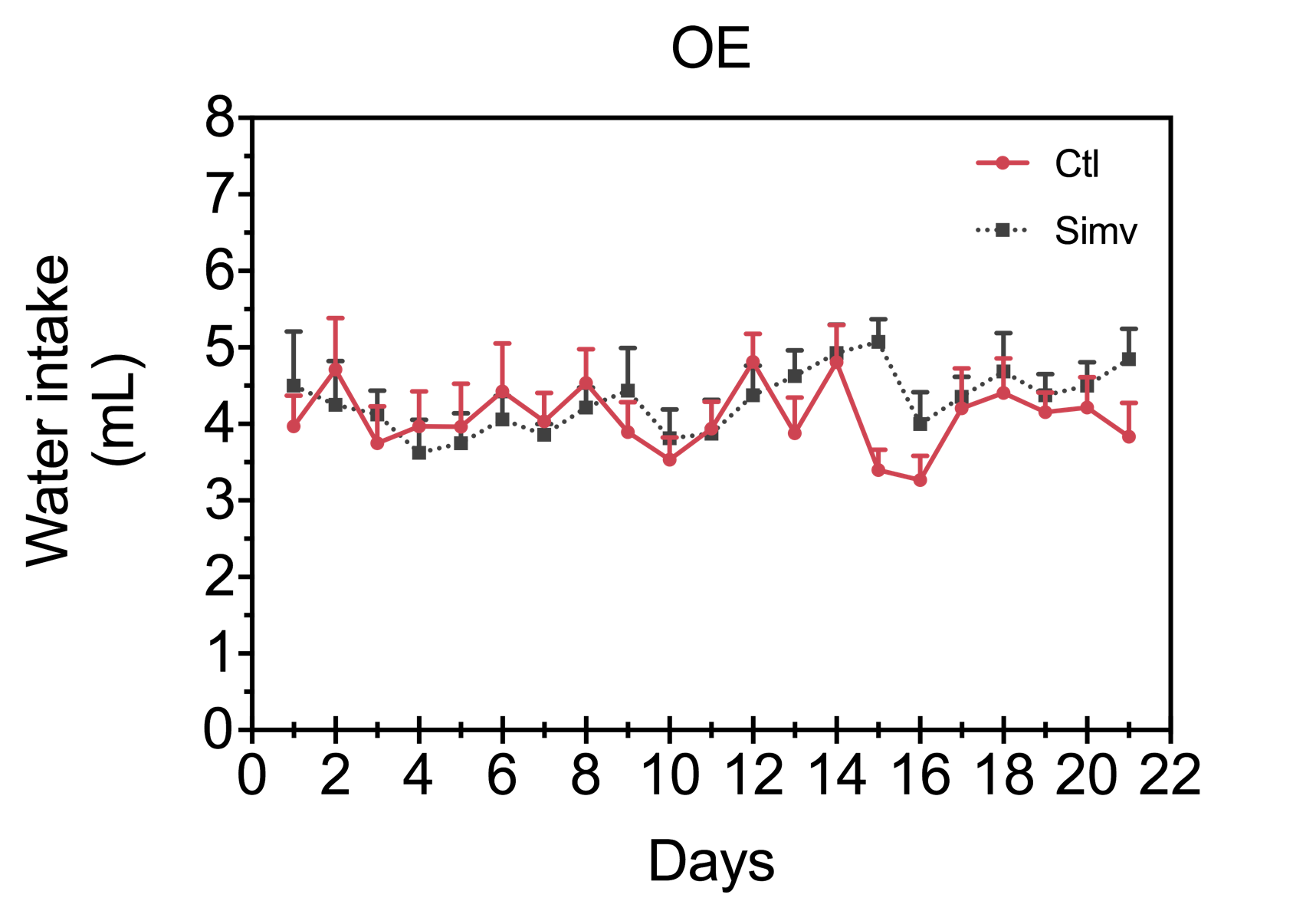

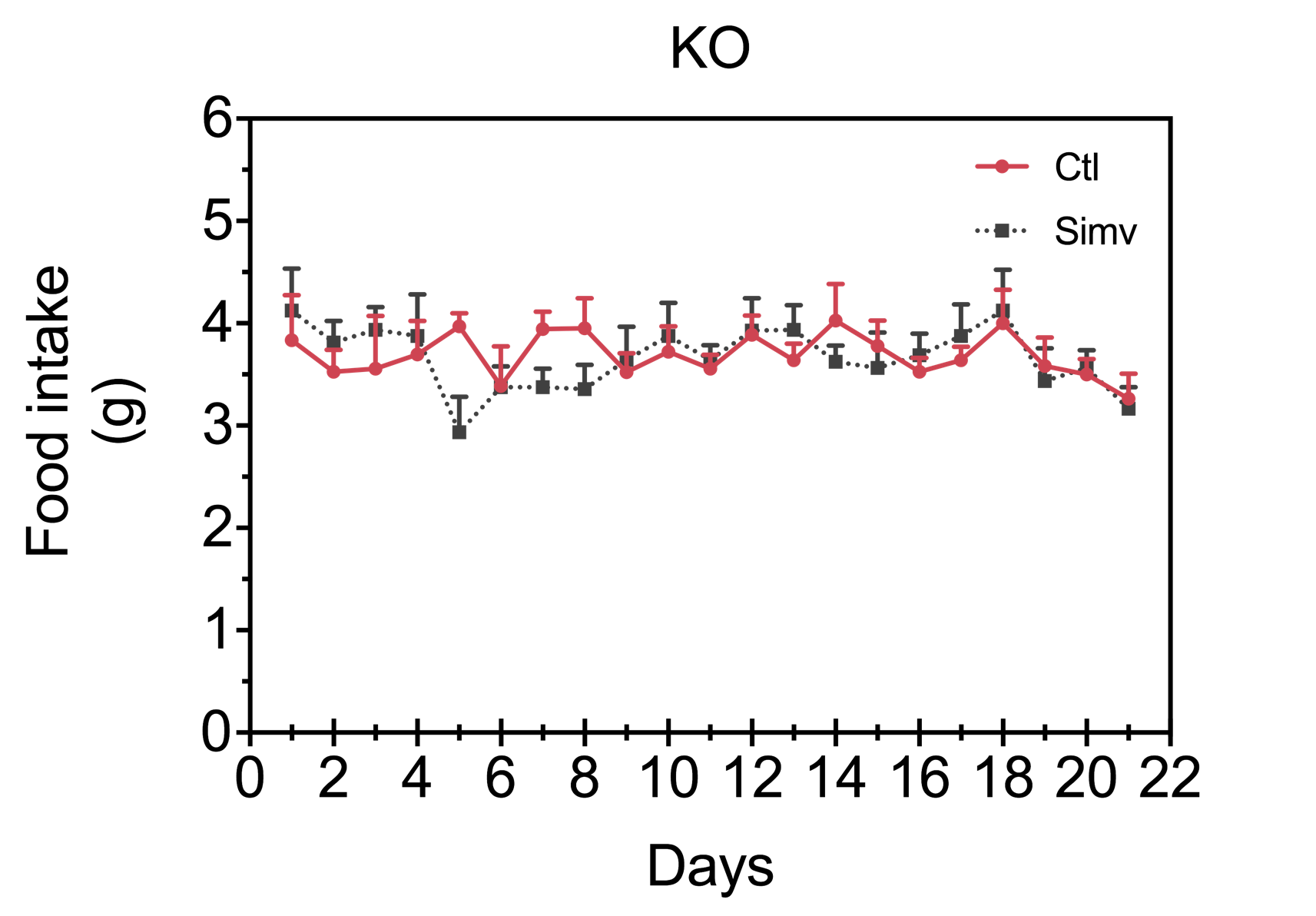

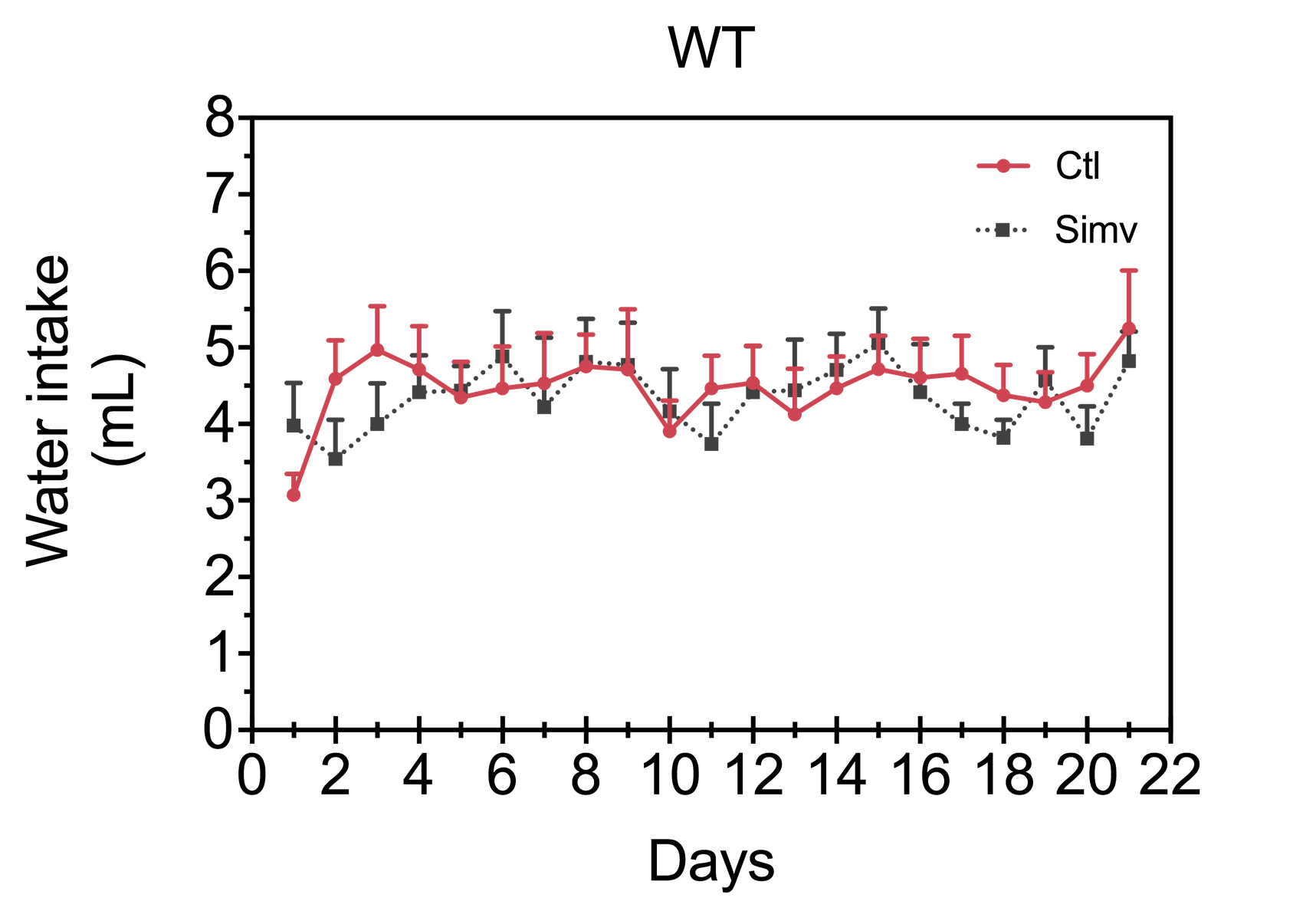


**A**

**C**

**B**

**D**

**F**

**E**

**Figure S2**. General physiological parameters. Physiological parameters, such as water and food intake, were monitored daily during the treatment. Food (A-C) and water (D-F) intake are presented as average daily values during the treatment For WT (A and D) KO (B and E) and OE mice (C and F). Data are presented as mean ± SEM of 10 animals per group. Ctl, control; KO, PGC‐1α knock‐out mice; OE, PGC‐1α overexpressing mice; Simv, simvastatin; WT, wild-type.
