# Supplementary figures and images for: Simvastatin Impairs Glucose Homeostasis in Mice Depending on PGC-1α Skeletal Muscle Expression
Source: Biomedicines. 2020 Sep 15;8(9):351. doi: 10.3390/biomedicines8090351 (PMC7555587; doi:10.3390/biomedicines8090351)

GLUT4

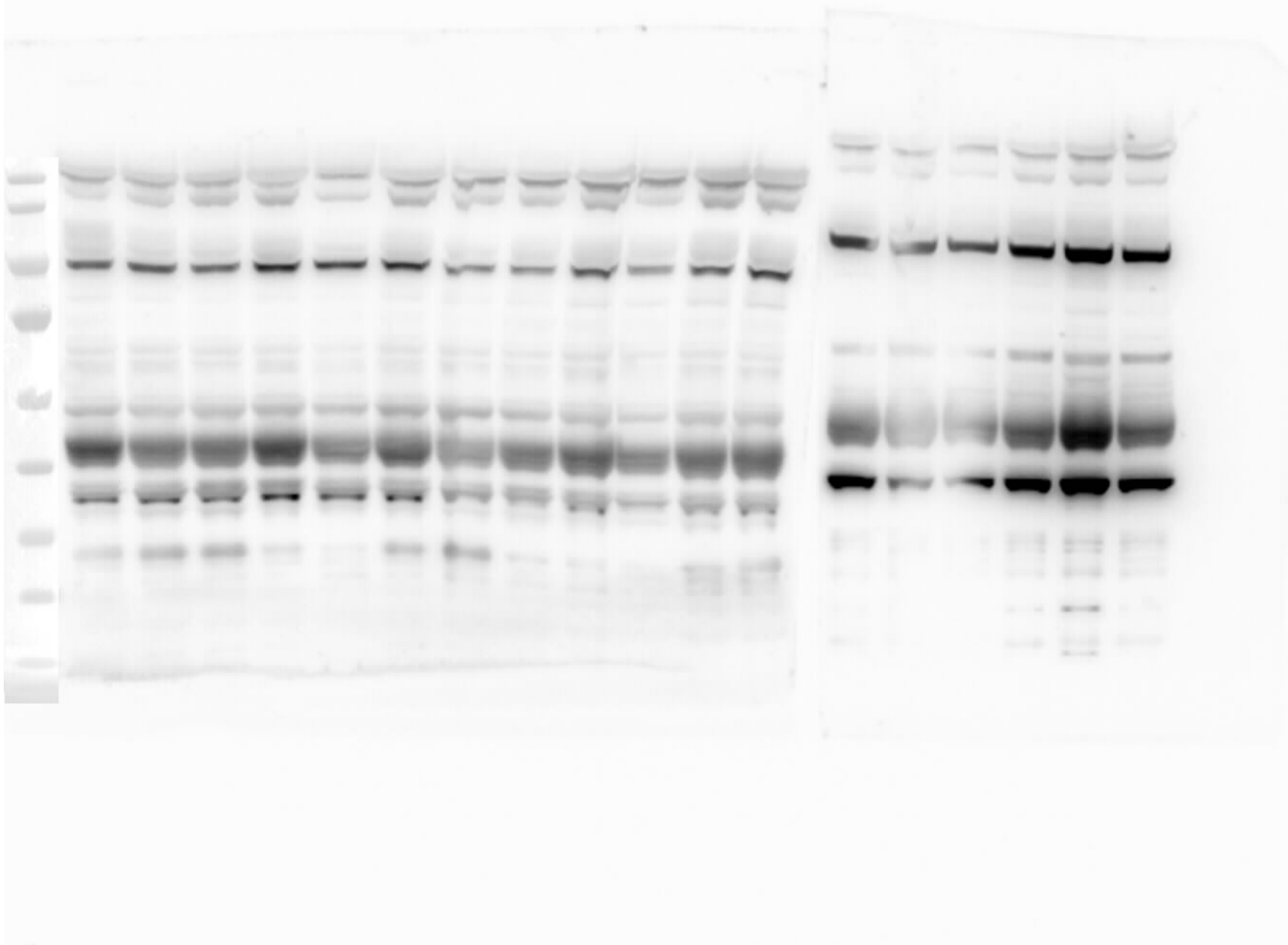

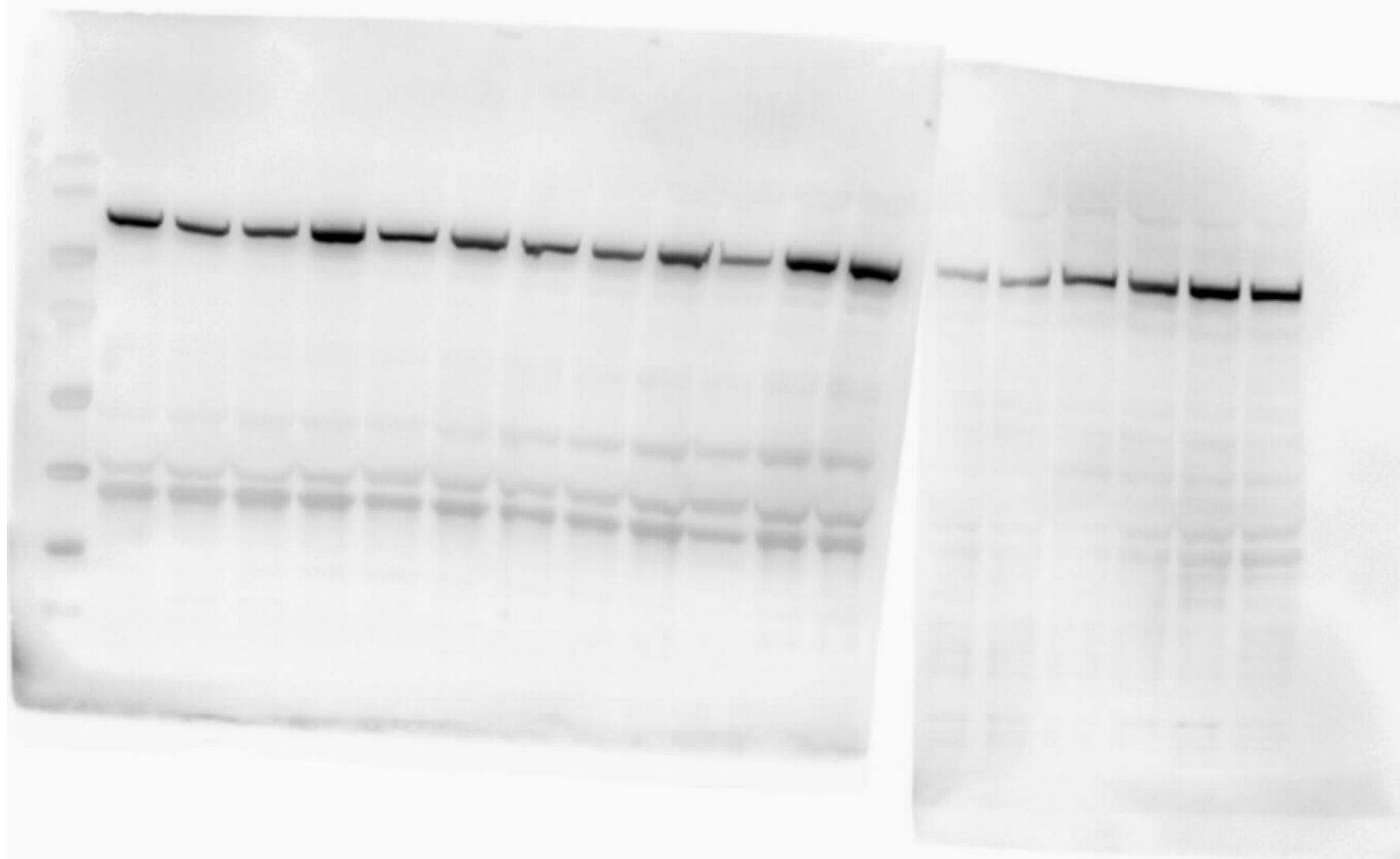

eEF2

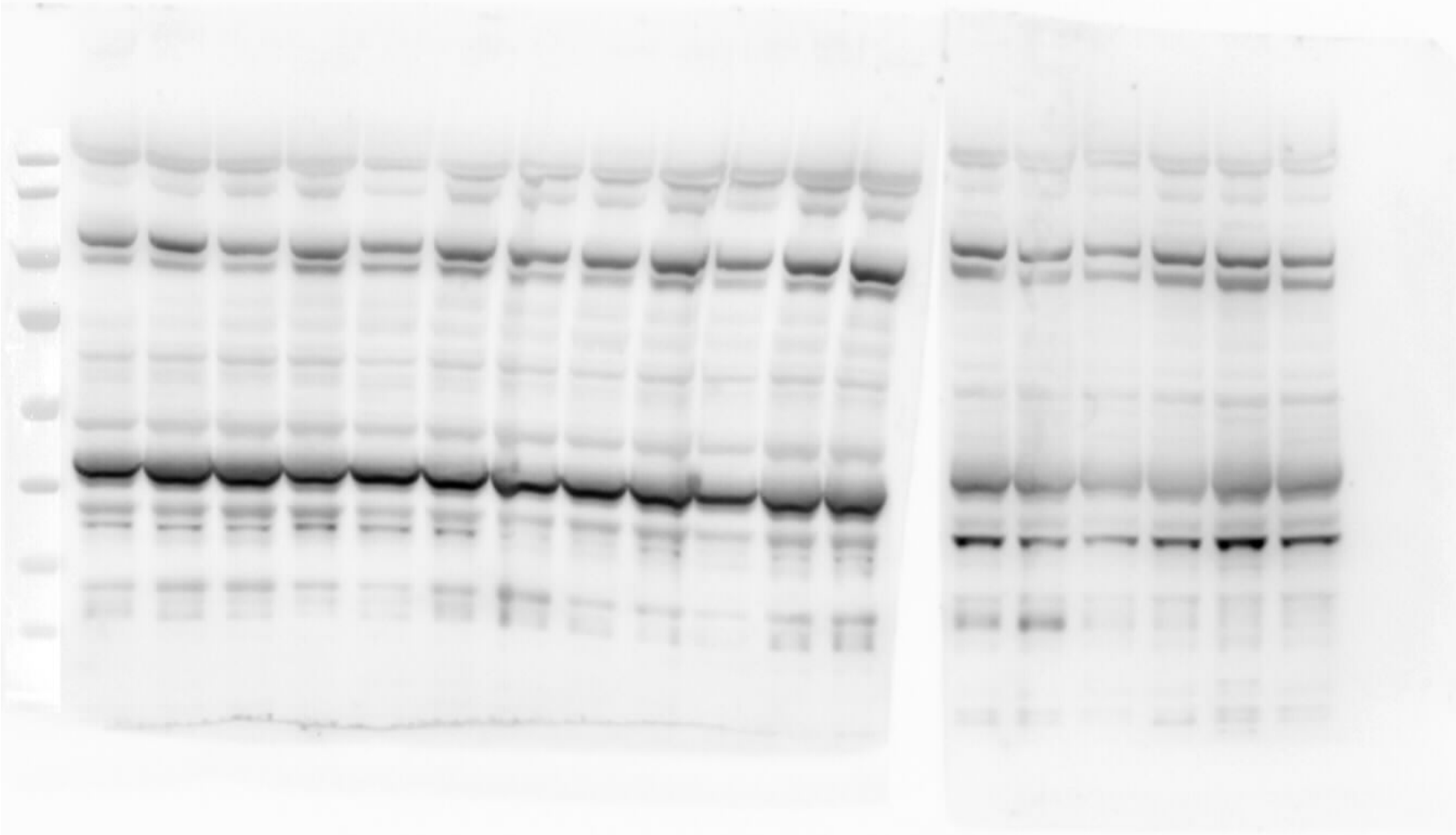

Supplement: Supplementary file 1 [file biomedicines-08-00351-s001.zip › Blot pictures Biomedicines_919087.pdf]
